# Hypofractionated Whole-Breast Irradiation Focus on Coronary Arteries and Cardiac Toxicity—A Narrative Review

**DOI:** 10.3389/fonc.2022.862819

**Published:** 2022-04-07

**Authors:** Camil Ciprian Mireştean, Roxana Irina Iancu, Dragoş Petru Teodor Iancu

**Affiliations:** ^1^Department of Medical Oncology and Radiotherapy, University of Medicine and Pharmacy Craiova, Craiova, Romania; ^2^Department of Surgery, Railways Clinical Hospital, Iasi, Romania; ^3^Oral Pathology Department, Grigore T. Popa University of Medicine and Pharmacy, Iasi, Romania; ^4^Department of Clinical Laboratory, St. Spiridon Emergency Hospital, Iaşi, Romania; ^5^Department of Medical Oncology and Radiotherapy Grigore T. Popa University of Medicine and Pharmacy, Iasi, Romania; ^6^Department of Radiation Oncology, Regional Institute of Oncology, Iasi, Romania

**Keywords:** cardiac toxicity, coronary arteries, hypo-fractionated radiotherapy, breast, chemotherapy, HF-WBI

## Abstract

Breast cancer is the most common cancer among women worldwide, which is often treated with radiotherapy. Whole breast irradiation (WBI) is one of the most common types of irradiation. Hypo-fractionated WBI (HF-WBI) reduces the treatment time from 5 to 3 weeks. Recent radiobiological and clinical evidence recommended the use of HF-WBI regardless of the age or stage of disease, and it is proven that hypo-fractionation is non-inferior to conventional fractionation regimen irradiation. However, some studies report an increased incidence of heart-related deaths in the case of breast irradiation by hypo-fractionation, especially in patients with pre-existing cardiac risk factors at the time of treatment. Due to the new technical possibilities of radiotherapy techniques, HF-WBI can reduce the risk of cardiac toxicity by controlling the doses received both by the heart and by the anatomical structures of the heart. The radiobiological “double trouble”, in particular “treble trouble”, for hypo-fractionated regimen scan be avoided by improving the methods of heart sparing based on image-guided irradiation (IGRT) and by using respiration control techniques so that late cardiac toxicity is expected to be limited. However, long-term follow-up of patients treated with HF-WBI with modern radiotherapy techniques is necessary considering the progress of systemic therapy, which is associated with long-term survival, and also the cardiac toxicity of new oncological treatments. The still unknown effects of small doses spread in large volumes on lung tissue may increase the risk of second malignancy, but they can also be indirectly involved in the later development of a heart disease. It is also necessary to develop multivariable radiobiological models that include histological, molecular, clinical, and therapeutic parameters to identify risk groups and dosimetric tolerance in order to limit the incidence of late cardiac events. MR-LINAC will be able to offer a new standard for reducing cardiac toxicity in the future, especially in neoadjuvant settings for small tumors.

## Introduction

The risk of cardiac toxicity developed at long intervals after irradiation of the whole breast for early-stage breast cancer in case of breast conservative surgery has been shown to be high, with a potential to cause mortality related to heart disease. Historical studies have shown an increased rate of adverse effects in patients treated for left breast cancer, a fact denied by new scientific evidence. It is considered that the risk of mortality for women treated for left breast cancer is equal to that of women treated for right breast cancer by whole breast radiotherapy (WBI). Hypo-fractionated adjuvant radiotherapy of the whole breast (HF-WBI) is also accepted for the adjuvant treatment of the left breast, with the results indicating a non-inferior loco-regional control and overall survival compared to the case when standard fractionation treatment was used. The HF-WBI is also associated with a 10-year heart rate toxicity equal to that of the control group in which 50 Gy was delivered in 25 daily fractions (1, 2).

Chen and collaborators demonstrated that, for a median follow-up of 14 years, there was no difference in the mortality of cardiac causes in women who were irradiated postoperatively with the conventional or hypo-fractionated regimen on the breast or chest wall. The authors did not report differences in comparative analysis of patient lots irradiated for incipient breast cancer after conservative surgery nor between cases treated for right breast or left breast cancer (3).

Different results have been reported by other authors in some studies, with hypo-fractionated WBI irradiation being associated with an increased risk of late cardiac toxicity. However, the results should be evaluated with caution because some studies reporting an excess of late cardiac events in HF-WBI groups have used conventional techniques and parasternal irradiation, leading to an increase in the doses received by the heart. However, there are factors that may significantly affect the results regarding the toxicities of the treatment and which were not taken into account in most retrospective studies. Thus, pre-existing cardiac diseases and diabetes are factors related to a patient’s medical history that seriously influence the late cardiac toxicity rates. Breast size and dosimetric parameters are also not recorded in most studies reporting a higher incidence of cardiac events in the HF-WBI group (4).

## HF-WBI in Clinical Practice

### HF-WBI and Historical Trials

The use of hypo-fractionation was initially evaluated in four phase III trials; the first of them was initiated in 1986 at the Royal Marsden Hospital and the Gloucestershire Oncology Center. The arm that included hypo-fractionation included 2 regimens (42.9 Gy in 13 fractions and 39 Gy in 13 fractions). The results show an increased rate of local relapses at 10 years in the arm that received 39 Gy in 13 fractions (14.8%), which was 2.7% higher than in the patient lot that received 50 Gy in 25 fractions (12.1%), but the hypo-fractionated regimen of 42 Gy in 13 fractions demonstrated the ability to reduce the rate of loco-regional recurrences (9.6%) at 2.7% compared to standard treatment (5).

A Canadian study by the Ontario Clinical Oncology Group reports differences between late-onset cardiac mortality for WBI-treated patients for the right and left breasts. The hypo-fractionated regimen was 42.56 Gy in 16 fractions, with a 10-year local recurrence rate of 7.5% in the 50-Gy arm and 7.4% in the hypo-fractionation arm and no significant differences in overall survival. The study demonstrates the feasibility of using HF-WBI. A limitation of this study is the use of information from administrative databases that raises suspicions about the correctness of coding and proper registration of risk factors and cause of death. The presence of large numbers of patients with preexisting risk factors associated with late cardiac toxicity could be associated with an increased number of deaths in the arm of patients irradiated with left breast cancer (6, 7).

START A trial was based on the fractionation schemes from the English trial group RMC/GOC, but the dose in one of the 2 arms of hypo-fractionation was reduced to 41.6 Gy. START A was initiated in 1999 with a design similar to the English RMC/GOC trial except for 42.9 Gy. The results were influenced by the use of 10 Gy in 5 fraction boosts in random cases and of the inhomogeneity of the treatment, with 35% receiving chemotherapy and 15% being operated by radical mastectomy. Trial START B was randomized between 1999 and 2001, with 2,215 patients in two groups: standard fractionation and HF-WBI (40 Gy in 15 fractions for 3 weeks), having the same drawback with the use of the 10-Gy boost in 5 fractions and inhomogeneity in the inclusion criteria. The difference between relapse rates at 10 years was 1.4% (3.8 *vs*. 5.2%) in favor of the arm with hypo-fractionated treatment (8). Given the uncertain evidence and the lack of long-term follow-up, the American Society of Radiation Oncology recommends caution in using hypo-fractionation, especially in the sense of avoiding the inclusion of the heart in the radiation field (9).

A study that enrolled 72,134 women diagnosed with breast cancer in 1976–2006 in Denmark and Sweden aimed to evaluate the long-term incidence of radiation-induced cardiovascular disease by comparing the risk of toxicity in the case of right and left breast irradiation. The ratio between the mean heart dose (MHD) for left and right breast irradiation was 2.33, and the highest incidence of cardiovascular events was pericarditis with a ratio of 1.61 and valvular diseases with a ratio of 1.54. Cardiac ischemic disease has been identified as a risk factor affecting toxicity. At the starting time of the historical hypo-fractionation studies, the adjuvant use of transtuzumab was not a therapeutic standard. The potential for cardiac events associated with anti-HER2 therapy is well known. At the same time, the increased life expectancy of patients treated with transtuzumab also increases the risk of cardiac death (10).

With a median follow-up of 13.2 years, a study that included 485 women treated by conventional fractionation whole breast irradiation (CF-WBI) and 5,334 women treated by HF-WBI under the age of 80 years found a difference in hospitalizations between the two groups of patients. Using a competing risk approach, the authors evaluated the cumulative cardiac morbidity of 10 and 15 years from radiotherapy for patients who received treatment on the right breast or the left breast. However, the inclusion criteria of the lots were not identical in the 2 arms. In addition to the fact that the group receiving CF-WBI included fewer mastectomy patients, it was more common in the HF-WBI group. In the CF-WBI patients, there was a higher prevalence of diabetes. The study does not reveal any difference in cardiac morbidity leading to hospitalization of cardiac causes between the 2 groups at 15 years of follow-up (11).

### HF-WBI—Risk Factors and Toxicity Profile

The patients’ preferences regarding the choice of conventional fractional or hypo-fractionated radiotherapy after breast-conserving surgery were investigated after a discussion with the oncologist. In total, 348 patients were included in a study, 259 patients of which were being treated by CF-WBI and 89 patients by HF-WBI. The investigators’ option was to offer HF-WBI to older patients who did not receive any adjuvant chemotherapy. The study focused only on evaluating acute toxicities considered similar in the two groups except breast pain. This was reported as being perceived as grade 2 only in the CF-WBI group. The authors recommend the use of hypo-fractionation as a method based on these data, the patients’ preference, and the lack of acute side effects (12).

The use of adjuvant radiotherapy concomitantly with anti-HER therapy for positive human breast cancer growth factor (HER2) is common, although data evaluating cardiac toxicity are relatively limited if irradiation is co-administered with trastuzumab in adjuvant settings. Data regarding the use of trastuzumab concurrently with HF-WBI are limited in HER2-positive breast cancer. Sayan et al. evaluated acute cardio-toxicity in HER2-positive breast cancer patients who received concurrent CF-WBI or HF-WBI radiotherapy by evaluating the left ventricular ejection fraction (LVEF). Cardiac toxicity was considered if an absolute decrease of LVEF was observed to be ≥10% below the lower limit of the normal value or ≥16% of the baseline value. The study concluded that there is no difference for decreasing the ratio of ejection fraction between the two groups, the median follow-up being 32 months (range, 13–90 months). The study included 100and 41 cases treated by CF- WBI and HF-WBI, respectively. With 7% reduction rate (LVEF) and without cases of congestive heart failure, the authors recommend a long-term evaluation of HF-WBI cardio-toxicity co-administered with transtuzumab. Even though in this study computer tomography (CT) simulation was used for all patients and the technique of blocked inspiration was used to reduce doses to the heart, the non-uniformity of the inclusion criteria was caused by the random use of boost in some patients, at the clinician’s decision. In a study evaluating the effect of the combination of hypo-fractionated irradiation and anti-HER2 treatment with transtuzumab, an asymptomatically significant rate of LVEF decline was found to be 7 and 5%, respectively, in groups of patients who received hypo-fractionated radiotherapy and conventional regimen. A variation of ≥10% from normal or ≥16% was considered significant cardiac impairment. The authors consider the difference between the rates of asymptomatic LVEF between the groups that received hypo-fractionated and conventional irradiation to be insignificant (13, 14).

HF-WBI is often used with caution in patients with large breasts, with an extended tumor bed, or for patients who have received chemotherapy due to the risk of possible late cosmetic effects of hypo-fractionation, probably determined by dose heterogeneity. Pectus excavatum or other congenital or acquired abnormalities of the rib cage and sternum alter the geometry of the thorax, and implicitly, the dosimetry may be associated with an increase in the dose received by the heart. Cardiomegaly involves not only an increase in the value of the cardiothoracic ratio above 0.5 but also the anatomical variations that increase the risk of the heart to be included in the irradiation field, not only being a potentiating factor of a possible cardiac toxicity but also a risk factor. As a pre-existing disease, there should be justifying caution in choosing hypo-fractionated radiotherapy. Patel and collaborators evaluated the effect of HF-WBI on patients with large breast volume, including 505 cases of which 502 were treated with HF-WBI, all with whole-breast clinical target volumes of ≥1,000 cm^3^. Using a 42.56-Gy hypo-fractionation scheme in 16 fractions in most cases plus a cavity boost of 10 Gy in 4 fractions, delivered both by three-dimensional conformal radiotherapy field-in-field technique and by intensity-modulated radiation therapy technique, the rate of acute grades 1, 2, and 3 dermatitis was 55.0, 40.8, and 3.4%, respectively. Three parameters were identified as predictors of severe dermatitis (a whole-breast clinical target volume of ≥1,500 cm^3^, body mass index ≥34, and V105 >10%). The authors recommend maintaining V105 <10% to maintain a grade 3 dermatitis rate of <2% when using hypo-fractionation for patients with large breasts. Using a spectrophotometric method, a study that included 24 patients identified primarily lighter skin color, breast size (small breasts), use of boost, and VMAT technique as risk factors associated with the risk of mild radiation-induced dermatitis. In order to evaluate the safety of the method, a randomized, multicenter study compared the irradiation with conventional CF-WBI fractionation in a total dose of 50 Gy/25 fractions + 10 to 14 Gy/5 to 7 fractions administered as a boost, with HF-WBI in a total dose of 42.56 Gy/16 fractions + 10 to 12.5 Gy/4 to 5 fractions for the boost. The study also showed a cosmetically favorable toxicity profile in cases that did not receive chemotherapy and only a lower increase in skin toxicity if hypo-fractionation was associated with chemotherapy. The authors strongly recommend the use of HF-WBI (13–17).

Without being the subject of this study, however, it should be noted that most trials evaluating the HF-WBI toxicity profile relate to cosmetic effects, especially acute dermatitis (17–20).

Evaluating a randomized unblinded interventional trial CF-WBI (50 Gy in 25 fractions for 5 weeks with a sequential boost of 10–14 Gy delivered in 5–7 fractions) and HF-WBI (46.56 Gy in 16 fractions for a week and a half plus a boost of 10–12.50 Gy in 4 to 5 fractions) followed by conservative breast surgery that included 287 cases with vastness greater than or equal to 40 years and TMN stage between 0 and II, Shaitelman and collaborators consider, after an evaluation of the data at 3 years after treatment, that HF-WBI is feasible both in terms of results and toxicity profile and large breasts. Chemotherapy and tumor bed boost are not factors that contraindicate hypo-fractionation (18, 19).

Another study that evaluated acute toxicities in a group of 140 patients with breast cancer who received adjuvant radiotherapy with both conventional fractionation and moderate hypo-fractionation showed much lower rates of acute dermatitis as assessed both by evaluation and reporting by the physician and patient, respectively, as well as by spectrophotometric evaluation in the case of HF-WBI. Not only were lower rates of acute dermatitis associated with hypo-fractionated radiotherapy but also hyperpigmentation and limitations of day-to-day activities were lower in the group of patients receiving HF-WBI (20).

## The Influence to Cardiac and Coronary Artery Toxicity of Systemic Oncological Treatment

The cardiac toxicity associated with the systemic treatments administered in the multidisciplinary treatment of breast cancer is well known, which is being associated with both conventional chemotherapy agents and with new innovative target therapies. All these treatments can cause different degrees of cardiac dysfunction. From a dose–effect relationship, type I cardiac toxicity is dose dependent and irreversible, and dose-independent cardiac toxicity is generally reversible. Pertuzumab, a humanized antibody targeting extracellular domain II of HER2, is currently combined with trastuzumab plus docetaxel, a standard first-line treatment in HER2-positive metastatic breast cancer, but the APHINITY trial confirms the value of adding transtuzumab for treatment the invasive HER2-positive early-stage breast cancer. In a total study group of 4,805, including both patients who received pertuzumab or placebo with a median follow-up of 74 months, they did not show elevated rates of cardiac adverse events, with their rate being maintained at <1%. It should be noted that the clinical benefit for pertuzumab addition was only obtained in patients with positive nodes (21).

The absence of changes in troponin-I (TnI) and brain natriuretic peptide (BNP) biomarkers in double-blocked HER2 in combination with taxanes demonstrates the safety of this treatment for cardiac toxicity in the absence of anthracycline therapy, confirming the safety of the transtuzumab–pertuzumab combination in the CLEOPATRA trial result. The final results of the CLEOPATRA trial showed only 1% rate of fatal treatment-related adverse events in the group receiving pertuzumab and 2% in the placebo group for a median follow-up of 8 years. There is 1 new case of congestive heart failure in the pertuzumab group (22–24).

Only one case of asymptomatic decrease in the left ventricular ejection fraction below 50% was identified in a batch of 23 cases of metastatic or recurrent breast cancer HER2+ who received treatment with radiotherapy for treatment with or for palliative care in combination with transtuzumab and pertuzumab. Concomitant dual HER2 blockade (pertuzumab and trastuzumab) with concurrent curative breast radiotherapy was not associated with cardiac toxicity in a study including 55 patients with a median follow-up of 4.1 years. The mean radiation dose was 50 Gy, and the mean decrease in left ventricular ejection fraction was -2.43% before and after chemotherapy and radiotherapy. However, Ito and collaborators report 2 cases in which dual HER2 blockade was associated with decreased ventricular ejection fraction stage in 2 Japanese patients aged 72 and 49 years after 6 and 13 cycles of transtuzumab, respectively (25–27).

The toxicity induced by anthracycline chemotherapy may be acute, manifested im-mediately after infusion of the drug or later, *i*.*e*., manifested at varying intervals from 1 to 10 years after treatment. From a clinical point of view, the toxicity of anthracyclines is manifested by thinning off of the wall of the left ventricle, with a gradual decrease of the ejection fraction of the left ventricle (28).

The observation of the cardiotoxicity of anthracyclines in children manifested by the onset of cardiomyopathy led to the conclusion that the cardiac impairment is determined by the death of the cardiomyocyte progenitor cells by apoptosis. A cumulative dose of 400 mg/m^2^ is associated with 3–5% risk of heart failure, with the risk increasing up to 26% at doses of 550 mg/m^2^ in the case of doxorubicin. The risk of toxicity is lower when liposomal doxorubicin and epirubicin are used. A history of radiation therapy and the concomitant use of cyclophosphamide, paclitaxel, and transtuzumab increase the risk of heart failure. Young and old age and pre-existing cardiac diseases are also factors with the potential to increase the rate of adverse effects (29, 30).

Alkylating agents, including cyclophosphamide, are associated with increased cardio-toxicity, the risk of heart failure being 7–28% at a dose of 150 mg/kg and 1.5 g/m^2^. The most common clinical manifestation is pericardial effusion, and the risk is increased for mediastinal irradiation and anthracycline treatment. An ifosfamide dosage >12.5 g/m^2^ is also associated with cardio-toxicity (31).

Antimicrotubic agents were associated with 1.7–5% risk of myocardial ischemia in the case of docetaxel and paclitaxel administration, respectively. Bradycardia without clinical significance in most cases is associated with a hypersensitivity reaction following histamine release. Paclitaxel also increases the risk of arrhythmias (32, 33).

Transtuzumab is associated with cardiomyopathy in 2–7% cases, with the potential for cardiotoxicity being increased to 13% risk if paclitaxel treatment is associated. The risk of heart disease can reach 27% if the treatment with anthracyclines is combined. The cardiac toxic effect of transtuzumab is not dose dependent but is strongly amplified by the association with anthracyclines that induce myocyte apoptosis. Studies in mice have shown a synergistic effect with potential for biventricular involvement of both the left ventricle and the right ventricle if the combination of doxorubicin with transtuzumab is administered (34).

A study that included 727 patients treated with anthracycline-based chemotherapy and taxanes and whole breast adjuvant 42.4 Gy radiotherapy in 16 daily fractions (2.65 Gy per fraction) evaluated the effect of transtuzumab addition regarding the degree ≥2 acute skin toxicity and cardiac toxicity, specifically LVEF. The results show a grade 1 (FEVS <60–50%) toxicity rate of 6.8% for patients treated with chemotherapy alone and 13.7% if transtuzumab was added to the regimen. In the case of grade 2 cardiac toxicity (LVEF <50–40%), the rate of toxicity was 1.1% *vs*. 2%, which was higher if transtuzumab was added to hypofractionated radiotherapy and chemotherapy. The authors note that acute cutaneous toxicity was not significantly higher with the addition of transtuzumab. A possible inhomogeneity of the inclusion criteria should also be mentioned; only patients with close or positive margins and those with grade 3 histological tumors received a radiation boost. The authors recommend cautionary measures regarding cardiac toxicity given the increased rate of cardiac toxicity ≥2 degrees in the group of patients who also had transtuzumab included in the therapeutic protocol but still considered transtuzumab with hypo-RT as well tolerated in breast cancer patients (35).

The relationship between cardiovascular effects and the risk of radiotherapy-related cardiac damage was observed both in the survivors of the atomic bomb and in patients who received radiation therapy for the treatment of peptic ulcer, with the risk of cardiac death being increased at >10 years after treatment. Irradiation produces microvascular damage in the case of myocardium and macrovascular damage of the coronary arteries. If in the case of the myocardium the effect appears at a relatively short interval from months to years but often remains asymptomatic due to the possibility of the myocardium to ensure its vascularization and the compensatory reaction to the reduction of the number of viable myocytes is fibrosis and hypertrophy, in the case of coronary artery disease, it is a late-onset effect, clinically manifested after years or decades of radiotherapy treatment. In the case of large vessels, irradiation causes pathological changes of the endothelium and triggers accelerated atherosclerosis. Epidemiological studies that evaluated cardiac mortality in patients treated with mediastinal radiotherapy for Hodgkin’s lymphoma revealed 2.7-fold increase in cardiac mortality, with the risk being increased by a radiation treatment history at a young age (36).

## Dose Volume Cardiac and Coronary Toxicity—Radiobiological Consideration

Historical clinical observations have shown high rates of cardiac mortality in patients irradiated by conventional radiation therapy for lymphoma in the mediastinum region. A higher mortality rate from causes other than those related to cancer has also been reported in old lots of patients who have been irradiated by conventional radiotherapy. In 1994, Cuzik et al. report, after analyzing data since 1975, that at that time the high mortality rate of all causes in 10-year survivors of breast cancer treatment was not significant, although there was a numerical difference in favor of untreated patients, with an excess of deaths occurring in older historical studies. The authors conclude that reducing the dose received by the heart is essential in reducing the cardiac toxicity of radiotherapy. In 1991, Emami and collaborators formulated the first recommendations based on the toxicity risk dependence according to the dose–volume parameters (48, 49).

Dosimetric recommendations were reviewed in the Quantitative Analyses of Normal Tissue Effects in the Clinic (QUANTEC) guidelines, in which retrospective studies involving more patients treated by 3D-conformal radiotherapy were analyzed. It was also mentioned that radiation therapy for Hodgkin’s lymphoma and breast cancer is associated with a much higher rate of cardiac mortality. The QUANTEC guidelines, although not capable of solving the problem of dose–volume constraints, offer some directions and dosimetric constraints that are respected in many radiotherapy centers.

The Quantec guidelines estimate a probability of <1% of cardiac mortality at 15 years for V25 <10% but also mentions the factors that increase the risk of synergistic toxic cardiac effects, especially when using a multimodal approach, including doxorubicin chemotherapy. The authors’ recommendation is to intensify research into the elucidation of the mechanisms and radiation doses associated with the increased rate of cardiac events (37).

Currently, all predictions for the risk of pericarditis associated with radiotherapy are based on the radiation dose delivered to the heart. The contouring guidelines do not define pericardium as an organ at risk (OAR), and dose recommendations in order to reduce the risk of pericarditis are not established. The dose conformation obtained by conventional radiotherapy with tangential fields makes the pericardium “a collateral victim of irradiation”, being the closest cardiac substructure in the vicinity of the treated target volume. Due to the steep dose gradient behind the target, the pericardium near the rib cage will receive a dose much higher than the rest of the heart. With the introduction of modern techniques of radiotherapy with modulated intensity that have been adopted in breast cancer, the dose distribution has been changed essentially. The current tendency is not only to delimit the whole heart as an OAR but also to delineate its anatomical sub-structures. Since 2010, Feng et al. published an atlas of the heart and cardiac substructure contouring. Douaneet and collaborators proposed 7 years later another atlas that allows the contouring of 15 different cardiac anatomical structures. The use of these atlases in future studies will facilitate the evaluation of dose–volume constraints with clinical toxicity potential. Most of the attention is focused on the coronary arteries and the heart chambers, but there are no recommendations regarding the contouring of the pericardium in atlases of heart contouring. Although radiation-induced pericarditis is rare in the age of modern radiotherapy techniques, it is often underdiagnosed, and the delineation of the pericardium as a risk structure and the identification of dosimetric constraints are necessary in this case, taking into account the unpredictable dose distribution associated with inverse planning techniques (38, 50, 51).

The advantage of modern techniques is brought by the image guidance of the treatment through the concept of IGRT based on the use of standard computer tomography (CT) simulation and the support of on-board imaging devices to perform precise positioning and to improve the ballistic accuracy of the irradiation beam. The administration of radiotherapy using the deep-inspiration breath-hold (DIBH) technique allows the heart to move downward and posteriorly in relation to the target volume of the breast or chest wall, thus increasing the degree of heart sparing (39, 51–55).

The variation of the doses received by the heart from one case to another is an unsolved problem in breast cancer radiotherapy, and the MHD is often used by the surrogate in the therapeutic decision and validation of a treatment plan. However, MHD may have similar values for a case when the whole heart is receiving a low homogeneous dose or a case when the pericardium in the vicinity of the target volume receives a high dose and the rest of the heart receives a lower dose of radiation. Of course, the adverse effects will be different in the two cases, and MHD cannot differentiate between these situations. The BACCART study by Jacob et al. highlights that MHD is not a sufficient dosimetric parameter to predict the individual dose received by the left ventricle and by the coronary arteries, especially to the left anterior descending coronary artery. MHD is not a sufficiently surrogate dosimetric parameter to estimate the risk of cardiac toxicity, with the results showing that although an MHD value <3 Gy was obtained, for 56% of patients treated for left breast cancer, the left anterior descending coronary artery (LADCA) could receive at doses >40 Gy. Dosimetric recommendations are mostly based on expert opinions and are still not sufficiently documented in order to make a prediction of radiation dose distribution for each case and implicitly of the cardiac toxicity risk. When choosing to use altered fractionation such as hypo-fractionation, the equivalent tumoricid dose and also the toxic dose received by the organs from vicinity of target volumes are approximated using the biological effective dose (BED) and can be calculated using the conventional linear–quadratic (LQ) model, which is considered accurate in estimating equivalent doses in case of dose values per fraction <10 Gy. BED differs for different α/β ratios due to tissue heterogeneity, being approximated with the value of 10 Gy for tumors and with the value of 3 Gy for healthy tissues. In reality, α/β differs significantly both for different histological types of tumors and for healthy tissues. In the case of the breast, α/β is considered approximately equal to 4 Gy, which justifies, from the radiobiological point of view, the use of hypo-fractionation. Tumor radio-sensitivity varies widely from case to case between patients who have the same histological type and the same tumor stage. Some authors recommend the use of isoeffect formula for biological effect estimation, assigning values α/β = 2, 3.5, and 5 Gy, respectively. The hot spots in the irradiated field become “hotter” as the physical radiation dose is transformed into BED, an effect called the “double trouble”. Thus, a more careful approach to treatment plans from the point of view of eliminating hot spots is needed both from the target volume and especially from healthy tissues. In order to evaluate the doses received by the heart and its structures, it is necessary to convert the actual doses into the dose equivalent to the radiation dose given in 2 Gy/fraction (EQD2) (40–43).

In the case of hypo-fractionated radiotherapy, a cause for concern is the effect generated by higher total doses associated with hot spots (higher doses per fraction partial volumes). Described as a risk factor for possible toxicities in conventional radiotherapy under the name of “double trouble”, the effect is associated with a much higher toxic risk in case of association with hypo-fractionation. In this case, the effect is known as “treble trouble”. More than 15 years ago, Jones and collaborators have proposed an adaptation of the LQ model, considered to be an ideal mathematical model, for various clinical situations in which tissue radio-sensitivity can be altered. The model proposed the addition of a BED equivalent or a mean dose-modifying factor (x) for each clinical and therapeutic feature that could decrease tissue tolerance to irradiation. Thus, in the case of adjuvant radiotherapy for breast cancer, *x*-value is estimated to 1,063 for subcutaneous fibrosis for cyclophosphamide, methotrexate, and fluorouracil (CMF) chemotherapy and radiotherapy and 1,033 for shoulder fibrosis in patients >60 years old and receiving lymph nodes. Equivalent to the effect in BED, CMF chemotherapy, surgery, and age were equated as effect with the addition of BED with 6.48, 17.73, and 3.61 Gy, respectively.

The hypo-fractionation of postoperative radiotherapy for breast cancer has been evaluated in a number of large randomized clinical trials, but concerns remain regarding late cardiac toxicity. A study evaluated the predictive ability of the LQ formalism for four evidence-based hypo-fractionation regimes regarding the dose received by the heart. Analyzing the treatment plans for 60 left breast cancer patients treated with WBI tangential fields, Appelt and collaborators corrected the doses to EQD2 using the LQ radiobiological model for five different fractionation schemes, (one conventional regimen and four hypo-fractionation regimens) using different values of α/β. The results of the radiobiological evaluation prove favorable results on hypo-fractionation for α/β = 3 Gy for the 40 Gy/15 fractions, 39 Gy/13 fractions, and 42.5 Gy/16 fractions regimes. The authors specify that if the heart can be protected by the dose prescribed for the tumor, the heart receives a lower dose even if it is considered α/β = 1 Gy. The 41.6 Gy in 13 daily fractions regimen does not achieve favorable results regarding heart sparing compared to the standard 50 Gy in 25 fractionation schemes. A study that includes 40 patients with early stages (T1/T2 + N0) of the left breast evaluated the effect of radiation therapy doses on the coronary arteries and heart in the case of adjuvant irradiation after a conservative breast surgery. The radiation doses received by the coronary arteries—LADCA, left circumflex coronary artery (LCx), whole heart, and heart cavities—right ventricle (RV), and left ventricle (LV) were delineated and subsequently dosimetrically evaluated. For all structures, the median dose and the average dose, V5 and V25, were evaluated. The authors identified LADCA as the structure that receives the highest average dose (24.02 ± 8.38 Gy) and proposed balancing the dose constraints between the different structures as a strategy to limit radiation-induced cardiac toxicity (44–47) Dose constraints for whole heart and heart anatomical structures for breast cancer radiotherapy recommended in clinical practice have been summarized in **Table 1**.

**Table 1:** Dose constraints for whole heart and heart anatomical structures for breast cancer radiotherapy (48–55).

Organ/structure/subvolume	Constraints (dose volume)	Recommendation (guidelines/author)
Heart	1/3 heart <60 Gy 2/3 heart <45 Gy 3/3 heart <40 Gy V25 <10% MHD <26 Gy V30 <46% Dmean <2.5 Gy Dmean <4 Gy V10 <30% (left breast) V10 <10% (right breast) V20 <5% (left breast) V20 = 0 (right breast) V20 = 10% V40 = 5% Dmax <40 Gy <1 Gy (right breast WBI) <2 Gy (left breast WBI) Use an alpha/beta ratio of 2.5 for the heart	Emami et al. Gagliardi et al. (QUANTEC) Piroth et al.; German Society of Radiation Oncology (DEGRO) RTOG 1005 hypofractionated trial Nielsen MH et al. Danish Breast Cooperative Group (DBCG) Smith et al.; American Society of Radiation Oncology
Left ventricle	Dmean <3 Gy Dmean <2.5 Gy V5 <17% V23 <5%	Piroth et al. DEGRO
Left anterior descending coronary artery	Dmax <20 Gy Dmean <10 Gy V30 <2%	DBCG DEGRO

## Ultra-hypofractionated Radiotherapy: Heading Towards a New Standard?

A multicenter, non-inferior, randomized, phase 3 trial compared the hypo-fractionation regimen proposed by the FAST-Forward trial (40 Gy in 15 fractions over 3 weeks) with 2 “ultra-hypo-fractionated” regimens regarding efficacy and toxic late effects, both evaluated at 5 years after treatment. Including 4,096 patients from 97 hospitals aged at least 18 years with invasive breast cancer (pT1-3, pN0-1, and M0) and treated with conservative surgery or mastectomy followed by radiotherapy, the phase III trial proposed 2 ultra-hypo-fractionated regimens (26Gy in 5 fractions and 27Gy in 5 fractions). A relative difference was found from the proposed FAST-Forward trial regimen of -0.3 and -0.7% for the treatment regimen of 27 Gy in 5 fractions and 26 Gy in 5 fractions, respectively, regarding the 5-year ipsilateral breast cancer relapse. Both moderate or marked clinically assessed toxicity and photographic evaluation of the breast were associated with a higher risk of late effects associated with the 27-Gy hypo-fractionation regimen in 5 fractions. The authors propose the ultra-hypo-fractionation scheme of 26 Gy in 5 fractions as a safe alternative to the moderate hypo-fractionation with 40 Gy in 15 fractions. After two more trials (Chinese and Danish) that consolidate evidence favorable to moderate hypo-fractionation, the results of the British trial proposing ultra-hypo-fractionation made Rodin and collaborators conclude that “standard” fractionation for is no longer the standard treatment for breast cancer. The Chinese trial, which included 734 patients, confirms that a moderate hypo-fractionated pattern of 43.5 Gy in 15 fractions over 3 weeks plus 8.7 Gy delivered as a boost is not inferior to the standard fractionation regimen plus a 10-Gy boost administered in 1 week. With a recurrence rate in the group with moderate hypo-fractionation of 1.2 *versus* 2% in the group with conventional fractionation and a similar rate of late toxicity in the two groups, the study achieves its goal. In the case of 2 to 3 degrees of acute skin toxicity, hypo-fractionation demonstrates not only non-inferiority but also an obvious benefit with a median follow-up of 73.5 months. The DBCG HYPO trial, a randomized phase III trial that included 882 patients >40 years of age who underwent conservative surgery for ductal carcinoma *in situ* (DCIS) and negative breast cancer nodes, demonstrated both non-inferiority of the moderate hypo-fractionation regimen (40 Gy in 15 fractions) regarding the risk of recurrence at 9 years and similar or even lower values of toxicity and risk of breast induration, compared with the results of the standard fractionation regimen (56–59).

Leap and Kirova point out the need to adjust the doses received by the heart and its anatomical structures in the context of the new ultra-hypo-fractionation schemes. Thus, the proposed dose limits for the standard fractionation regimen may be a trap; the doses proposed by the consensus of DEGRO breast cancer experts (MHD <2.5 Gy, mean left ventricle dose <3.0 Gy, V30 Gy for the left anterior descending coronary artery <2%) also requires accurate evaluation in clinical trials that include a 26 Gy/5 fraction regimen to limit the risk of late toxic cardiac effects, in the context of which the exact value of the alpha/beta parameter is unknown for the heart and its anatomical structures (51, 60).

## Strategies to Limit Heart Dose Irradiation: Deep Inspiration Breath Hold and Prone Treatment Position

The DIBH technique is part of the strategies designed to minimize the radiation dose received by the heart using its distance from the chest wall by inflating the lung. The patient will maintain deep inspiration during the delivery of radiation and thus will limit both movements of the target volume, avoiding irradiation with higher doses, of the heart and especially of LADCA. Comparing dosimetrically normal breath irradiation with the DIBH technique for post-mastectomy left breast radiotherapy, Darapu et al. obtained favorable results for V5, V10, V25, V30, and Dmean of heart and also for the ipsilateral lung in the case of DIHB. The authors also maintain the disadvantages of the technique (it is time consuming and requires patient cooperation, team training, and careful treatment setup). The technique is considered advantageous both in the use of conventional fractionation and in hypo-fractionation or stereotactic irradiation with a single dose for thoracic and upper abdominal tumors (61, 62).

Even if it clearly demonstrates a dosimetric benefit with a supposed effect in reducing the late cardiac toxicity profile, DIBH does not solve the source of uncertainty generated by heart contractions during treatment. A study that included 20 patients evaluated the displacement of LADA using the contouring of this structure as a risk organ based on systolic and diastolic phase using computed tomography-based coronary angiography with retrospective electrocardiographic gating. All cases were evaluated for the DIHB technique. The average displacement to the posterior area of the treatment field was 2.3, 2.6, and 2.3 mm in left–right and anteroposterior direction, respectively. Although the DIHB technique is not associated with a considerable displacement of the heart during contractions, LAD position variations may differ greatly between the systolic and diastolic phases from one patient to another. The authors recommended maintaining a distance of ≥5 mm between the LAD and the irradiation field edge in order to reduce the risk of coronary events associated with radiotherapy (63).

Longer-duration deep inspire breath-hold for a time of up to 3 min using voluntary hyperventilation, high nasal prong flow, or even continuous positive airway pressure could maximize heart protection. However, with the new irradiation techniques, the delivery time of the dose in case of irradiation of the chest wall and nodal levels may require up to 5 min. Identifying a solution to increase the level of heart protection is a challenge for future investigation. DIHB VMAT planning showed a reduction from 5.4 to 3.6 Gy of MHD, and D2% to the heart was reduced from 19.3 to 13.4 Gy compared to VMAT-free breath (FB-VMAT) treatment plans. In the case of LADA, the average value of Dmean was also reduced from 6.9 to 3.9 Gy, and D2% was reduced from 19.5 to 9 Gy by using the DIHB technique associated with VMAT left breast irradiation (64, 65).

The use of prone position *versus* supine position in radiotherapy of the left breast is still controversial in terms of the benefit of this position for heart sparing. A support vector machine algorithm was chosen to identify parameters that would be suggestive of the need for supine position re-planning, with 198 left side breast patients being included in the study. Cases associated with ≤0.1 cc of the heart included in the irradiation field were excluded, with the algorithm being applied only to plans that included >0.1 cc of the heart in the target volumes isodose. Heart orientation, heart–tumor distance, and lung volumes included in the irradiation field were identified as significant parameters to form the basis of an exclusion algorithm regarding the need for a re-simulation CT planning in supine position and also for the acceptance of the treatment plans in prone position. A dosimetric study proposed by Wang et al., including 116 cases of left side breast cancer treated with the DIHB technique, demonstrated the prone position benefit in 61% of cases compared to supine DIHB, with the benefit being greater for high pendulousness and moderately large breasts. Prone DIHB had the best results in terms of heart and lung sparing for left side WBI when the technique was compared with shallow breathing (SB), supine DIBH, and prone SB (42, 66–73).

If in the case of the reduced dose received by the lung the prone position proved to be 100% beneficial, for MHD, in 15% of cases there was a benefit for supine position. However, the results of this historical study, which included 400 patients—60% of whom met the criteria for inclusion in the evaluation, demonstrate the superiority of prone position in heart sparing by reducing the average in-field heart volume by 7.5 cm^3^, representing a median reduction of 85.7% (74).

## MR-LINAC—State-of-the-Art Technology for Reducing Cardiac Toxicity

Soft tissue contrast obtained with MR-LINAC machines, a hybrid unit that combines magnetic resonance imaging with linear accelerator, has demonstrated the ability to reduce the high doses received by radiosensitive structures, including cardiac anatomical structures. A study that explored the capacity of MR-LINAC evaluated the mean and maximum (0.03 cc) doses for the heart, LADA, and left ventricle and also the volume that receives at least 5 Gy (V5) of the left ventricle. The optimized plans demonstrated the ability of the technique to reduce MHD by up to 2.5 Gy and the left atrial mean dose by up to 1.2 Gy. Mention should also be made of an increase of >10 Gy in 4 cardiac substrates in one case in the evaluated group. All evaluated cardiac structures were able to benefit from a reduction in D0.03cc and mean dose by rescheduling based on 0.35-T MRIs acquired on an MR-LINAC to optimize CT-based plans to limit cardiac toxicity (75).

When MR-LINAC is used for partial breast irradiation (PBI) in neoadjuvant settings, a better delimitation of GTV may result in a reduction in margins and therefore a limitation of toxicity. For *in situ* tumors, the ability of MR-LINAC to delimit/irradiate the gross tumor more precisely is obvious, and a higher reduction of setup errors can be observed in both cases of neoadjuvant and adjuvant settings. Comparatively evaluating the doses received by OARs for PBI planned in prone and supine position using MR-LINAC, Koerkamp et al. identified a benefit in reducing V5Gy to ipsilateral long for prone position, with the MHD results being similar in both positions for treatment (76–78).

## Conclusions

The use of HF-WBI radiotherapy has now become a standard in adjuvant treatment after breast-conservatory surgery, although the preliminary results show the feasibility and technique in terms of benefit in loco-regional control and toxicity studies are needed to evaluate the long-term and very-long-term implications, especially for patients who have been irradiated for left breast cancer. It is also necessary to elucidate the mechanisms of action of the new oncological therapies and the potential synergistic interactions with chemotherapy and radiotherapy. Studies that will include the newly considered radiosensitive anatomical structures of the heart, especially the ventricles and coronary arteries, and will correlate toxicity with the doses received by them and with biological and genetic factors related to the patient and the treatments administered will lead to new dose-volume recommendations and to the development of radiobiological models for estimating the risk of toxicity with clinical applicability. The still unknown effects of small doses spread in large volumes on lung tissue may increase the risk of second malignancy, but they can also be indirectly involved in the later development of a heart disease. The changes in conclusions in historical series will probably be accounted for by changes in radiotherapy technique, *e*.*g*., use of cobalt therapy, and improved treatment planning due to CT scanning with lung density correction and use of intensity modulation techniques to improve dose uniformity. These will have reduced cardiac dose exposure. However, on the use of hypo-fractionated and especially ultra-hypo-fractionated techniques, associated with a much richer spectrum in systemic therapies, all in the context of considerable improvements in the prognosis of breast cancer, special attention should be paid in the future to limiting the toxic effects of radiotherapy on the heart and coronary arteries. MR-LINAC will be able to offer a new standard for reducing cardiac toxicity in the future, especially in neoadjuvant settings for small tumors.

## Author Contributions

All authors contributed to the article and approved the submitted version.

## Conflict of Interest

The authors declare that the research was conducted in the absence of any commercial or financial relationships that could be construed as a potential conflict of interest.

## Publisher’s Note

All claims expressed in this article are solely those of the authors and do not necessarily represent those of their affiliated organizations, or those of the publisher, the editors and the reviewers. Any product that may be evaluated in this article, or claim that may be made by its manufacturer, is not guaranteed or endorsed by the publisher.
